# Pseudocyst of Ectopic Pancreas of the Duodenal Wall Masquerading as Malignant Cystic Tumor of Pancreas

**DOI:** 10.4103/1319-3767.56101

**Published:** 2009-10

**Authors:** Dharamanjai K. Sharma, Shaleen Agarwal, Ravindra K. Saran, Anil K. Agarwal

**Affiliations:** Department of Gastrointestinal Surgery, Govind Ballabh Pant Hospital and Maulana Azad Medical College, New Delhi, India; 1 Department of Pathology, Govind Ballabh Pant Hospital and Maulana Azad Medical College, New Delhi, India

**Keywords:** Ectopic pancreas, pseudocyst, chronic pancreatitis, duodenum

## Abstract

We report a patient who underwent pancreaticoduodenectomy for a cystic lesion in the region of the pancreatic head and duodenum. Preoperatively, we had suspected a malignant lesion; however, it turned out to be ectopic pancreatic tissue in the duodenal wall, with the changes of chronic pancreatitis and pseudocyst formation. With this report we seek to highlight the rarity of this particular pathologic combination and the difficulties in its correct preoperative diagnosis and management.

Ectopic pancreas is an uncommon condition. It is usually found in the gastrointestinal tract, with 24–38% being seen in the stomach, 9–36% in the duodenum, and 0.5–27% in the jejunum.[1–3] It is usually asymptomatic, being found only incidentally at laparotomy. Even when symptomatic, preoperative diagnosis is difficult. We present a patient who underwent pancreaticoduodenectomy for a cystic lesion in the region of the pancreatic head and duodenum that appeared malignant on preoperative investigations. Histopathology revealed pseudocyst formation and the changes of chronic pancreatitis in an ectopic pancreas in the duodenal wall. This pathologic combination is rare, difficult to diagnose preoperatively, and presents problems in management.

## CASE REPORT

A 50-year-old male patient, a chronic alcoholic as well as a chronic smoker, presented with 1½ months' history of moderately severe nonradiating pain localized to the upper abdomen. He had no history of acute pancreatitis, jaundice, anorexia, weight loss, or other such illness. There was no history of any similar illness in the family. The pulse rate was 86/min, blood pressure 126/80 mm Hg, and respiratory rate 16/min. His temperature was normal, he weighed 58 kg, and his height was 165 cm. General and abdominal examination revealed no abnormalities. Standard laboratory investigations, including hemoglobin, blood counts, blood sugar, and renal and hepatic function tests, were within normal limits. Serum amylase was 32 IU/l (normal: 30-100 IU/l).

**Figure 1:**
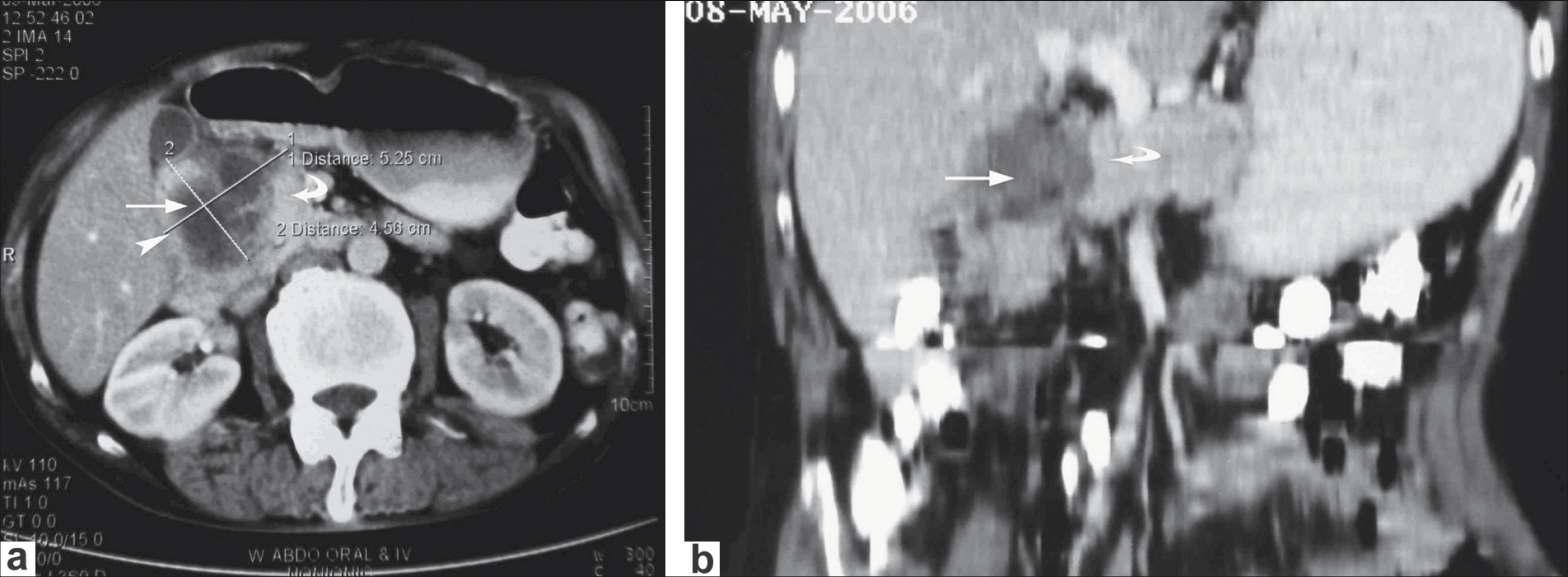
(a) CECT scan images showing the lesion (arrow) with cystic areas in relation to duodenum (arrowhead) and head of pancreas (curved arrow) and; (b) CECT scan images showing the lesion (arrow) with cystic areas in relation to duodenum (arrowhead) and head of pancreas (curved arrow)

Abdominal ultrasonography (USG) revealed two hypoechoic lesions (25 mm × 12 mm and 39 mm × 55 mm in size) in relation to the pancreatic head region. Endoscopic USG (EUS) confirmed these findings. Abdominal contrast-enhanced computed tomography (CECT) scan of abdomen showed a mass in relation to the duodenum and the head of the pancreas. The mass had central cystic areas and there was periportal and precaval lymphadenopathy [Figure 1a and b]. The rest of the pancreas appeared normal in size, shape, and attenuation. Attempts to acquire USG- and EUS- guided fine needle aspiration cytology (FNAC) from the lesion failed, but CECT-guided FNAC showed atypical cells, suggesting the possibility of malignancy. Cancer antigen (CA) 19-9 was 16.7 U/ml (normal range: 1.9-24 U/ml).

Based on the CECT and FNAC findings, the patient was provisionally diagnosed as having a malignant cystic tumor of the head of the pancreas and posted for surgery. Diagnostic laparoscopy revealed no evidence of metastases. On exploration, a hard mass in the pancreatic head region was noted, with multiple lymph nodes in the vicinity. The rest of the pancreas was firm. In view of the imaging and operative findings, as well as the inability to rule out malignancy, Whipple pancreaticoduodenectomy was carried out. The patient recovered well and was discharged on the 12^th^ postoperative day; he has been under follow-up for 6 months now.

**Figure 2:**
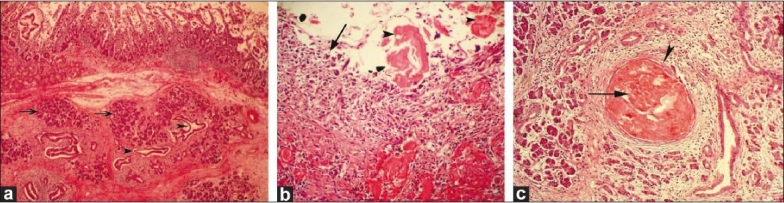
(a) Slide showing presence of pancreatic acini (arrows) and ducts (arrowheads) within the submucosa of duodenal wall (H and E, ×100), (b) Slide showing wall of pseudocyst (arrow) without lining epithelium and consisting of inflammatory granulation tissue and inspissated hyaline proteinaceous material (arrowhead) in the lumen. (H and E, ×400), (c) Presence of chronic pancreatitis and inspissated material (arrow) in dilated atrophic duct (arrowhead) in pancreatic head area. (H and E, ×200)

Histopathological examination (HPE) showed unexpected and uncommon findings [Figure 2a–c]. Gross examination revealed a 6 × 5 cm mass involving the duodenum, with two cystic areas (approximately 1 × 1 cm each). The duodenal mucosa over the mass was intact. The mass turned out to be ectopically located pancreatic tissue with the changes of pancreatitis and cyst formation. The cysts were confirmed to be pseudocysts since they did not have any lining epithelium, thus excluding the possibility of their being retention cysts. The pancreaticoduodenal interface did not show collection of fluid or acute inflammatory changes, but there were foci with the features of chronic inflammation in the form of fibrosis. The native pancreas showed changes of chronic pancreatitis, which was less marked than that seen in the ectopic pancreatic tissue. The bile duct and main pancreatic duct in the resected specimen were not dilated, but the smaller pancreatic ducts showed changes characteristic of chronic pancreatitis [Figure 2c]. There was no evidence of malignancy and the lymph nodes showed only reactive changes.

## DISCUSSION

Most patients with ectopic pancreas are asymptomatic or have nonspecific symptoms, such as abdominal pain or discomfort, nausea and vomiting, gastrointestinal bleeding, etc. Symptoms may be due to the mass effect of the ectopic tissue or because of its involvement in a pathologic process that may also involve the normally placed pancreas (e.g., acute pancreatitis,[4] pancreatic abscess,[1] etc.). Pseudocyst formation in the setting of chronic pancreatitis can occur in an ectopic pancreas but it has only been rarely reported when ectopic pancreas is located in duodenal wall.[5] To the best of our knowledge, this is only the second case report of chronic pancreatitis with pseudocyst formation in duodenal ectopic pancreas. However, because of its ability to masquerade as malignancy, this condition is of clinical significance and must be considered in the differential diagnosis.

Abdominal contrast-enhanced computed tomography (CECT) scan of abdomen showed a mass in relation to the duodenum and the head of the pancreas.

The previously reported patient also presented with abdominal pain and was diagnosed as malignancy (duodenal sarcoma with cystic degeneration) on preoperative imaging; that patient too underwent pancreaticoduodenectomy.[5] Another reported patient had pancreatitis and pseudocyst formation in ectopic pancreas situated in the jejunum; the features clinically mimicked jejunal diverticulitis.[6]

Cysts can form in ectopic pancreas due to retention of secretions because of the absence of communication between the glandular tissue and the bowel lumen. However, true pseudocyst formation in ectopic pancreas is rare.[7] Cyst amylase content does not differentiate pseudocyst from retention cyst, being raised in both conditions.[7] In the present case, the lesions were considered to be pseudocysts as they were entirely lined by granulation tissue and lacked lining epithelium. Pseudocyst formation might have resulted from an earlier episode of acute on chronic pancreatitis.

Although chronic pancreatitis was present in both ectopic as well as native pancreas, it was minimal in the latter. This preferential involvement of the ectopic pancreas might have been due to the absence of a draining duct, causing chronic pancreatitis by way of mechanisms similar to those occurring in obstructive chronic pancreatitis; this obstructive element was absent in the native pancreas.

Duodenal wall cystic lesions can also arise from enterogenous duplication, retention cysts in Brunner glands, and cystadenomas,[8] and these conditions constitute the differential diagnoses.

There are three histologic subtypes of ectopic pancreas: Type I (similar to normal pancreas, with acini, ducts, and islet cells); type II (predominantly containing acini, no islet cells); and type III (predominantly ducts, no islet cells).[5] The present case was a type II ectopic pancreas, with chronic pancreatitis and pseudocyst formation.

The management of ectopic pancreas remains controversial. It has been reported that in 61% of cases the symptoms are attributable to the ectopic pancreas itself.[9] However, it has also been reported that in most cases the abdominal discomfort does not arise from the ectopic pancreas and can often be controlled with medical treatment alone.[10] These conflicting findings have led to difficulty in deciding the ideal treatment for this condition. It has been recommended that if symptoms persist—and after other diseases like peptic ulcer, gastroesophageal reflux disease, or biliary tract disease have been ruled out—surgical extirpation should be performed. If the benign nature of the lesion can be ascertained, asymptomatic patients can be kept under observation. Excision is performed if the nature of the lesion is uncertain. Surgery was indicated in our patient since he was symptomatic and malignancy could not be ruled out on the preoperative workup.

To summarize, in this article we describe a rare case of an ectopic pancreas in the duodenum, with chronic pancreatitisand pseudocyst formation. We highlight the difficulty in making a preoperative diagnosis even with the use of advanced diagnostic modalities and the limitation of FNAC in this setting.
